# Effects of Health Risk Assessment and Counselling on Fruit and Vegetable Intake in Older People: A Pragmatic Randomised Controlled Trial

**DOI:** 10.1007/s12603-020-1373-9

**Published:** 2020-04-25

**Authors:** A.M. Herghelegiu, K.M. Wenzel, A. Moser, G.I. Prada, C.R. Nuta, Andreas Ernst Stuck

**Affiliations:** 1 National Institute of Gerontology and Geriatrics “Ana Aslan”, Bucharest, Romania; 2 Department of Geriatrics, Inselspital, University Hospital Bern, and University of Bern, Bern, Switzerland; 3 Epidemiology, Biostatistics and Prevention Institute, University of Zurich, Zurich, Switzerland

**Keywords:** Fruit and vegetable intake, fibre intake, Mediterranean diet, health counselling, health risk assessment

## Abstract

**Objectives:**

Interventions to increase fruit and vegetable intake among community-dwelling older people have shown mixed effects. We investigated whether an intervention based on an initial multidimensional health risk assessment and subsequent physician-lead nutrition counselling has favourable effects on dietary intake among community-dwelling older people.

**Design:**

Randomised controlled trial comparing the intervention versus usual care.

**Setting and participants:**

Non-disabled persons aged 65 years or older at an ambulatory geriatric clinic in Bucharest, Romania, allocated to intervention (n=100) and control (n=100) groups.

**Intervention:**

Participants received a computer-generated health profile report based on answers to a health risk assessment questionnaire, followed by monthly individual counselling sessions with a geriatrician on topics related to health promotion and disease prevention, with a special focus on adequate fruit and vegetable consumption.

**Measurements:**

Fruit and vegetable intake at baseline and at 6-month follow-up.

**Results:**

At baseline, fruit and vegetable intake was below the recommended five portions per day in most study participants (85% in the intervention group, and 86% among controls, respectively). At six months, intake increased in the intervention group from a median of 3.8 to 4.6 portions per day, and decreased in the control group due to a seasonal effect from a median of 3.8 to 3.1 portions per day. At six months, fruit and vegetable consumption was significantly higher among persons in the intervention group as compared to controls (median difference 1.4 portions per day, 95% confidence interval 1.1–1.7, p<0.001).

**Conclusion:**

Personalised food-based dietary guidance, delivered as part of multidimensional preventive health counselling during geriatric clinic visits, results in relevant improvement of fruit and vegetable intake in community-dwelling older adults.

## Abbreviations

CI: confidence interval
IADL: IQR, interquartile range
HRA: health risk assessment
OR: odds ratio

## Introduction

High fruit and vegetable intake is associated with favourable health-related outcomes in older people. It reduces mortality and morbidity related to cardiovascular disease, cancer, diabetes mellitus, and other conditions in adult persons, including in subsamples of older persons (1, 2, 3, 4). Many health promotion programs include recommendations for increasing fruit and vegetable intake, or promote diets that include fruit and vegetable intake, such as the Mediterranean or DASH (Dietary Approaches to Stop Hypertension) diet (5, 6, 7). Recent research suggests benefits that are even more pronounced for older people given the impact of dietary intake on frailty (8, 9, 10, 11, 12), cognitive function (13, 14, 15), functional status (16), faecal continence (17), and other age-related health conditions (18, 19, 20).

Unfortunately, intake of fruit and vegetables continues to be below recommended levels in older people, and efforts for improvement have shown mixed results and demonstrated that it is challenging to change nutrition behaviour in older people (21, 22, 23). Nutrition counselling programs usually result in small effects, or effects may not be persistent because these programs are not integrated into usual care, resulting in organisational or financial barriers for widespread dissemination (24).

A promising approach might be the integration of specific health promotion and disease prevention topics into ambulatory clinical care, thereby giving the physician the opportunity to specifically address and emphasise nutrition counselling as part of usual care. In this approach, nutrition-counselling takes into account possible interactions of nutrition with health, physical activity, medication use or other factors in old age, potentially enhancing counselling effects. Previous studies of such multidimensional programs demonstrated that this is feasible, but it remains unclear to what extent this approach actually results in a relevant improvement in the dietary intake of older people (25, 26, 27, 28). We evaluated the effects of a novel intervention based on multidimensional health risk assessment and subsequent physician-lead counselling on fruit and vegetable consumption in older community-dwelling persons. We hypothesised that this model of intensive, individualised intervention in a geriatric care setting might substantially improve fruit and vegetable consumption in people of advanced age.

## Methods

### Study organisation

This study is part of the RAHEO (Medical Risk Assessment and Health Education in Older People) trial examining the impact of health risk assessment (HRA) and specialised geriatric counselling on physical activity, nutrition, and preventive care uptake in community-dwelling older people. The methods and base-line results of this study have been previously published (29). The field phase of this study took place at the Ambulatory Clinic of the National Institute of Gerontology and Geriatrics “Ana Aslan,” Bucharest, Romania between May 2014 and February 2015.

### Selection and randomisation of the participants

Consecutive patients 65 years of age and older, who were referred from general practitioners, were considered for study inclusion. Patients who met any of the following criteria were excluded from study participation: moderate to severe dementia, severe disability (needs help with one or more basic activities of daily living), terminal illness, major surgery within the last three months, not living in catchment area (Bucharest and within 2 to 4 hours travel time), living in nursing home, inability to speak/understand Romanian language, and inability or unwillingness to complete the pre-randomisation questionnaires. Persons who refused to give informed consent were also excluded.

Subsequent allocation to study groups was carried out using a randomisation procedure designed by the independent study centre in Bern, Switzerland using a computer generated 1:1 ratio allocation sequence in a block randomisation process. Participants were allocated to study and control groups in equal numbers using sealed opaque envelopes supplied by the study centre.

### Data collection

Prior to randomisation, a trained interviewer conducted face-to-face interviews with participants using a standardised pre-randomisation questionnaire that assessed self-perceived health status, self-reported health measurements, dependency in basic activities of daily living, and chronic conditions. Subsequently, participants were asked to complete a self-administered version of a Health Risk Appraisal (HRA) for Older Persons questionnaire. This questionnaire had originally been developed for use in multiple European countries in English and German languages (30). For the purpose of the present study, a Romanian version of this questionnaire was prepared, using translation, backward translation and cultural adaptation procedures. The HRA for Older Persons questionnaire consists of questions designed to identify potential health and disability risks, as well as lifestyle hazards (30). The questions are grouped into several domains evaluating functionality and mobility, medication use, sensory impairments, presence of pain, psychosocial health and well-being, social network, tobacco and alcohol consumption, level of physical activity, nutritional intake and preventive care.

Fruit and vegetable intake was measured by a six-item food-frequency questionnaire which is part of the self-administered HRA for Older Persons questionnaire (30). This dietary intake questionnaire is based on the validated rapid food screener to assess fat and fruit and vegetable intake based on dietary intake data from the Second U.S. National Health and Nutrition Examination Survey (31, 32). Specifically, the dietary intake questionnaire contains pictograms and text descriptions for defining the size of a portion in each of the six categories: fruit or vegetable juice: 200 ml of unsweetened juice; fruit and berries: one fruit weighing about 120 g or about one handful of berries; salad: one portion weighing about 120 g corresponding to a small plate or bowl; vegetables: about 120 g corresponding to half a cup; vegetable soup: one bowl; and tomato sauce: half a cup.

Consumption frequency in the last week was documented with six response categories: “none in the last week”, “once or twice in the last week”, “three to six times per week”, “once a day”, “twice a day”, or “three or more times a day”. Based on these answer categories, individual items were combined to calculate a composite score indicating the overall number of fruit and vegetable portions consumed per day per study participant.

At six month follow-up, participants were asked to complete the HRA for Older Persons questionnaire again, including the same six questions on fruit and vegetable intake. Participants completed the questionnaires by themselves on site, or with help from a research assistant, if requested. All research assistants were blinded to participants' treatment allocation status. The completed questionnaires were scanned and sent to the study centre in Bern for double data entry.

### Intervention

An individualised computer-generated feed-back report based on the participant's answers to the baseline HRA for Older Persons questionnaire was provided to the geriatrician counsellor and the patient at the initial counselling session. For fruit and vegetable consumption, the algorithm determined whether the patient adhered to the five portions per day recommendation. If yes, the report encouraged the participant to continue with this healthy nutrition habit. If not, the report stated: “You reported that you eat less than 5 portions of fruit and vegetables a day. Watch out! Something can be done for your health here. Try to get more fruit and vegetables into your diet. Fruit and vegetables provide vitamins and support the gut.”

The initial counselling session with the geriatrician lasted approximately 30 minutes. Health counselling started with recommendations related to physical activity, followed by nutrition counselling, and advice on additional topics, such as preventive care. As part of nutrition counselling, the geriatrician explained changes in nutritional needs with advancing age, and emphasised health benefits of optimal nutrient intake. Participants with satisfactory fruit and vegetable consumption were encouraged to continue to follow their dietary habits. Specific recommendations to encourage balanced, healthy nutrition were provided. Examples of typical local nutrient rich, high-fibre foods were offered, and participant preferences, as well as level of income, were taken into account. Participants were asked about specific conditions that might require a modified diet (e.g., irritable bowel syndrome, constipation, diabetes, hyperuricemia, gastro-oesophageal reflux disease) and current medications that might require avoidance of particular foods (e.g., statins, antibiotics); nutritional recommendations were made accordingly. The geriatrician also made referrals for gastroenterological, diabetes and metabolic workups, or for stomatological assessment, as needed. Participants were actively involved in the process of learning and were encouraged to discuss their diet and the recommendations they received with family members and friends. They were also encouraged to ask questions and to discuss any concerns related to their dietary choices with the health specialist. Participants were advised to plan their meals for several days in advance, and to make shopping lists to facilitate allocation of financial resources and ensure consistency of healthy eating. At the end of the initial counselling session, participants received a written summary report with recommendations, key points of the discussion, and the date and time of the next appointment.

Participants attended monthly follow-up sessions with the geriatrician for six months. Each session lasted between 15 and 30 minutes. Participants were asked about their progress in following the nutritional recommendations and reasons for non-adherence were explored. The geriatrician reinforced the importance of following the nutritional recommendations and modified recommendations based on a participant's progress. The last counselling session at six months included additional reinforcement to encourage continued adherence with the recommendations. Participants allocated to the intervention group received the intervention, plus usual care from their general practitioners during the entire study period.

Participants allocated to the control group received usual primary care, and did not receive the intervention.

### Statistical analysis

The estimated sample size to detect a 20% increase in the proportion of persons adhering to recommended health behaviour was 200, based on an expected drop-out rate of 10%, a two-sided alpha of 0.05, a power of 0.8, and a control group prevalence of adherence to recommended health behaviour of 60% (based on a power analysis for two-sample proportions test). All analyses were conducted according to a detailed analysis plan (29).

For the purpose of this study, the primary outcomes at six-month follow-up were the proportion of persons adhering to the recommended ≥5 portions of fruit and vegetable intake per day, and the amount of fruit and vegetable intake at six-month follow-up. Secondary outcomes were defined as the number of portions reported for each of the six specific fruit and vegetable categories. In addition, we identified the proportion of persons increasing fruit and vegetable intake by comparing response categories between baseline and six-month follow-up for each of the six categories. Group differences were tested by a chi-squared test for binary variables, and by a Kruskal-Wallis test for continuous variables. For binary variables, we report odds ratios (OR) with 95% confidence intervals (CI) from logistic regression models as effect measure. If no effect measure was estimable due to no event outcome, we report a p-value from an exact Fisher test for group comparison. For continuous variables, we report median group differences from quantile regression models (33) with 95% CI. All p-values are two-sided. Possible selection bias was assessed by an inverse probability of censoring weighting approach (34, 35). Statistical analyses were performed in R V.3.1.1 (R Project, University of Vienna, Austria).

Exploratory subgroup analyses were also performed to investigate the effects of the intervention on specific subgroups of the study population. However, because of sample size limitation and the post hoc approach of these subgroup analyses, no measures of effect were attempted and only descriptive results are presented.

## Results

**Figure 1:**
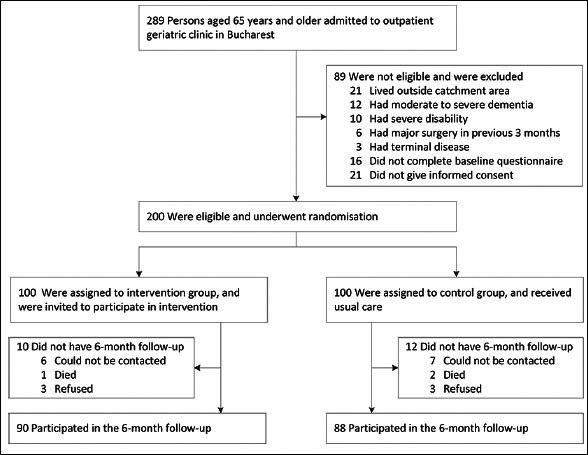
Flow diagram

Figure 1 depicts the CONSORT flow diagram indicating the number of persons excluded due to non-eligibility. Overall, 200 persons were included in the study and allocated to intervention (n=100) and control groups (n=100). There were no notable differences in baseline characteristics between the two groups (Table 1). Study participants had a mean age of 75 years, and the majority were highly educated, non-disabled women. At baseline, self-reported fruit and vegetable intake was low in both intervention and control groups, with the vast majority of participants not adhering to the recommended intake of five or more portions per day (85% among persons in the intervention group, and 86% among controls). In both groups, participants reported consuming a median of 3.8 portions of fruits and vegetables per day, consisting mostly of fruits, berries, and vegetable soup. About one third of participants reported that they intended to increase their consumption of fruits and vegetables in the future.

**Table 1:** Characteristics of study participants in control and intervention group at baseline

**Characteristic**		**Control (n=100)**	**Intervention (n=100)**
General characteristics			
	Age (years) — median (IQR)	75.0 (10.1)	74.8 (9.9)
	Women — n (%)	72 (72.0%)	77 (77.0%)
	Income < 848^a^ — n (%)	19 (19.0%)	25 (25.0%)
	Education: high school or more — n (%)	92 (92.0%)	93 (93.0%)
	Living alone — n (%)	9 (9.0%)	7 (7.0%)
	Number of chronic conditions — median (IQR)	6.0 (2.0)	6.0 (2.0)
	Fair or poor self-perceived health — n (%)	57 (57.0%)	65 (65.0%)
	Limitation in instrumental activities of daily living — n (%)	10 (10.0%)	14 (14.0%)
Overall fruit and vegetable intake			
	Number of fruit/ vegetable portions per day — median (IQR)	3.8 (1.1)	3.8 (1.4)
	Consumption of ≥5 fruit/ vegetable portions per day — n (%)	14 (14.0%)	15 (15.0%)
	Intention to increase fruit/ vegetable intake — n (%)	32^b^ (32.3%)	33 (33.0%)
Consumption of individual fruit/ vegetable items — n (%)			
	Fruit juice: consumption of ≥1 portion per week	28 (28.0%)	26 (26.0%)
	Fruit and berries: consumption of ≥1 portion per day	62 (62.0%)	69 (69.0%)
	Salad: consumption of ≥1 portion per day	1 (1.0%)	5 (5.0%)
	Vegetables: consumption of ≥1 portion per day	22 (22.0%)	26 (26.0%)
	Vegetable soup: consumption of ≥1 portion per day	42 (42.0%)	43 (43.0%)
	Tomato sauce: consumption of &≥1 portion per week	31 (31.0%)	36 (36.0%)


a. Corresponds to average pension per month expressed in local currency RON (36). An amount lower than this amount is considered very low income; b. n=99 due to missing information in one participant.

Eighty-eight participants in the control group and 90 participants in the intervention group completed the six-month follow-up (Figure 1). Reasons for non-completion were loss to follow-up with unknown survival status (control group n=7, intervention group n=6), death (control group n=2, intervention group n=1), and withdrawal of informed consent (control group n=3, intervention group n=3).

At six-month follow-up, fruit and vegetable consumption was higher in the intervention group (4.6 portions per day) as compared to the control group (3.1 portions per day) (Table 2). The median difference between the two groups was 1.4 portions per day (p<0.001) and was due to two factors. First, fruit and vegetable intake increased among participants in the intervention group from 3.8 portions per day at baseline (Table 1) to 4.6 portions per day at six-month follow-up (Table 2). Second, fruit and vegetable intake decreased among participants in the control group from 3.8 at baseline (Table 1) to 3.1 portions per day at follow-up (Table 2). This decline is explained by a seasonal effect (baseline data were collected between May and July while six-month follow-up data were collected between November and February).

**Table 2:** Fruit and vegetable consumption at six-month follow-up

**Parameter**	**Control group, n=88**	**Intervention group, n=90**	**OR or median difference (95% CI)**	**p-value**
Primary outcomes				
Number of portions of fruit/ vegetable per day- median (IQR)	3.1 (0.3)	4.6 (1.6)	1.4 (1.1, 1.7)	<0.001
Consumption of ≥5 portions of fruit/vegetable per day- n (%)	1 (1.1%)	35 (38.9%)	55.4 (7.4, 415.8)	<0.001
Individual fruit/ vegetable items: consumption at six months — n (%)				
Fruit juice: ≥1 portion per week	8 (9.1%)	38 (42.2%)	7.3 (3.2, 16.9)	<0.001
Fruit and berries: ≥1 portion per day	59 (67.0%)	88 (97.8%)	21.6 (5.0, 94.1)	<0.001
Salad: ≥1 portion per day	0 (0%)	1 (1.1%)	n.a.	&0.999^a^
Vegetables: ≥1 portion per day	42 (47.7%)	89 (98.9%)	97.5 (13.0, 731.0)	<0.001
Vegetable soup: ≥1 portion per day	17 (19.3%)	10 (11.1%)	0.5 (0.2, 1.2)	0.13
Tomato sauce: ≥1 portion per week	11 (12.5%)	51 (56.7%)	9.2 (4.3, 19.5)	<0.001
Increase in consumption from baseline to six-month follow-up^b^ — n (%)				
Increase in fruit juice consumption	1 (1.1%)	18 (20.0%)	21.8 (2.8, 166.9)	0.003
Increase in fruit and berries consumption	12 (13.6%)	53 (58.9%)	9.1 (4.3, 19.0)	<0.001
Increase in salad consumption	0 (0.0%)	10 (11.1%)	n.a.	0.002
Increase in vegetable consumption	34 (38.6%)	76 (84.4%)	8.6 (4.2, 17.6)	<0.001
Increase in vegetable soup consumption	13 (14.8%)	5 (5.6%)	0.3 (0.1, 1.0)	0.05
Increase in tomato sauce consumption	5 (5.7%)	29 (32.2%)	7.9 (2.9, 21.6)	<0.001


OR, odds ratio; CI, confidence interval; IQR, interquartile range; n.a., not applicable, a. p-value from exact Fisher test; b. An increase was defined as a change from a lower to a higher self-reported intake category between base-line and six-month follow-up.

In parallel, the proportion of participants who adhered to the recommended daily intake of five portions of fruit and vegetables increased among participants in the intervention group (from 15.0% at baseline to 38.9% at follow-up), and decreased among participants in the control group (from 14.0% at baseline to 1.1% at follow-up). Secondary detailed analyses revealed older people in the intervention group increased consumption of fruits, berries, and vegetables (Table 2). The intervention also resulted in a small increase in salad, tomato soup, and fruit juice consumption, but it had no effect on vegetable soup consumption. The sensitivity analyses conducted to assess possible selection bias due to missing six-month follow-up information (inverse probability of censoring weighting) revealed similar results for the primary outcome (median difference of number of portions of fruit/ vegetables per day 1.4 (1.1, 1.7; 95% CI, p<0.001). None of the study participants experienced any harm or other unintended effects related to the study.

**Table 3:** Number of fruit/vegetable portions per day in various subgroups at six-month follow-up

**Definition of subgroup**^a^	**Control group**	**Intervention group**
Subgroup of participants with income < 848 RON^b^ at baseline (n=40) — median (IQR)	4.5 (1.4)	3.1 (0.2)
Subgroup of participants without intention to increase fruit and vegetable intake at baseline (n=120) — median (IQR)	4.5 (1.5)	3.1 (0.3)
Subgroup of participants with fair/poor self-perceived health at baseline (n=103) — median (IQR)	4.5 (1.6)	3.1 (0.3)


IQR, interquartile range; a. Denominators in subgroups include participants for whom follow-up data were available; b. Corresponds to average pension per month expressed in local currency RON (36). An amount lower than this amount is considered very low income.

## Discussion

This innovative intervention of multidimensional HRA combined with geriatrician-lead counselling for physical activity, nutrition and preventive care resulted in a substantial increase in fruit and vegetable intake in a sample of Romanian community-dwelling older people. At baseline, the vast majority (over 80% of study participants) did not adhere to the recommended intake of five portions of fruit and vegetables per day. Following the intervention, participants in the intervention group consumed approximately 1.5 additional portions of fruits and vegetables per day, as compared to control group participants. This increase in fruit and vegetable intake was observed even for participants with low income, low initial intention to improve nutrition behaviour, and poor self-reported health.

The effect observed in our study corresponds to an increased intake of approximately 180 g of fruits and vegetables per day, and is likely clinically relevant. Based on estimates from the National Health and Nutrition Examination surveys an increase of 100 g of fruit and 100 g of vegetables per day reduces mortality risk from stroke by 10%, and 14% respectively (3). In comparison, other intervention models have shown no, or only modest, improvement in fruit and vegetable intake (21, 22, 23, 24, 25, 26, 27, 28). It is likely that a combination of factors contributed to these positive findings. First, by having a geriatrician provide nutrition counselling, participants may have viewed the individualised recommendations as health-related prescriptions, and this may have encouraged adherence. Second, counselling was individualised, taking into account each person's overall health status, coexisting factors, and preferences, and included specific recommendations tailored for the situation of each participant based on the HRA approach. Third, counselling was repeated at monthly intervals, thereby providing an opportunity for the participant and geriatrician to discuss progress with implementation of recommendations, problem-solve barriers, and revise recommendations, if needed. Finally, the intervention was highly structured, including written material that was provided to the participant. We previously demonstrated that this intervention approach is highly successful for increasing physical activity among older persons (29). Our current findings indicate that this approach is also highly effective for improving nutrition intake among older people.

We found strong seasonal variations in nutritional intake over the course of the study. Previous studies have also demonstrated that nutrition intake might vary by season, most likely in regions with relevant differences between summer and winter weather (37, 38, 39). In earlier times, fruit and vegetable products were not easily available in the Bucharest area during the winter. Although many products are now available all year round, older persons are apparently still less likely to buy fruit and vegetable products in the winter.

The present study has several limitations. First, generalisability is limited in a single-site study with a study population consisting of patients referred to a geriatric clinic, although this study provides evidence that older persons with different characteristics might benefit from such an intervention. Second, outcome data are based on self-report, and no observational or weekly diary data were collected. We tried to minimise bias by blinding outcome assessors to treatment allocation status of study participants and by using a validated food frequency questionnaire. Third, an overall measure of fruit and vegetable intake has limitations, since it derives one composite score based on six selected fruit and vegetable items with approximate numbers of portions per day. Fourth, our study examines fruit and vegetable intake, and does not address other relevant aspects of nutrition, such as fat or protein intake. Finally, the present study had no follow-up beyond six-months. Consequently, we do not know whether these intervention effects persisted over time.

## Conclusion

This study provides evidence that HRA combined with a geriatrician-lead nutrition intervention can improve nutrition intake in older persons. The study also demonstrates the feasibility of implementing a multidimensional health promotion approach with a focus on multiple aspects of health behaviours and preventive care within a geriatric outpatient clinic. Given the limitation of a single-site study using self-reported outcome data, additional research is warranted to confirm our findings. We believe that this nutrition-based intervention is a promising approach for addressing the nutritional needs of older persons and dissemination of this approach could have positive impacts on cardiovascular disease burden, functional status, cognition, frailty, and other health-related outcomes in old age.

## Trial Registration

International Standard Randomized Controlled Trial Number: ISRCTN11166046

## Electronic supplementary material

CONSORT 2010 checklist of information to include when reporting a randomised trial
